# Risk factors for postpartum hemorrhage in twin pregnancies with cesarean section

**DOI:** 10.3389/fmed.2023.1301807

**Published:** 2024-01-09

**Authors:** Yehui Lan, Anjian Xu, Xinyue Lu, Yujia Zhou, Jianping Wang, Ying Hua, Ke Dong

**Affiliations:** Department of Obstetrics and Gynecology, The Second Affiliated Hospital of Wenzhou Medical University, Wenzhou, China

**Keywords:** postpartum hemorrhage (PPH), twin pregnancies, cesarean section, risk factor, intertwin delivery time

## Abstract

The rates of twin pregnancies and cesarean section have increased in recent years, and both of them are at high risks of postpartum hemorrhage (PPH). However, few studies have concentrated on the risks of PPH in twin pregnancies and cesarean deliveries. In this study, we aimed to identify the risk factors for PPH among twin-pregnant women with cesarean section. This was a retrospective observational study including 1,649 women with twin pregnancies delivered by cesarean section from 2016 to 2022 in the Second Affiliated Hospital of Wenzhou Medical University, China. The eligible women were divided into PPH group (*n* = 116) and non-PPH group (*n* = 1,533) according to the blood loss after delivery within 24 h. The baseline maternal and perinatal characteristics were compared between the two groups. Logistic regression analysis was conducted to identify the potential risk factors for PPH. We found nulliparity, assisted reproductive technology (ART) usage, preeclampsia or HELLP syndrome, placenta previa, placenta accreta and general anesthesia were more common in PPH group than non-PPH group (*P* < 0.05). Women in PPH group had higher maternal body mass index at delivery and higher combined birthweight of the twins than non-PPH group, but had lower parity (*P* < 0.05). Seven independent risk factors for PPH were identified after logistic regression analysis: ART usage (OR 2.354 95% CI 1.357–4.083, *P* = 0.002), preeclampsia or HELLP syndrome (OR 2.605, 95% CI 1.471–4.616, *P* = 0.001), placenta previa (OR 7.325, 95% CI 3.651–14.697, *P* < 0.001), placenta accreta (OR 6.296, 95% CI 1.316–30.12, *P* = 0.021), thrombocytopenia (OR 1.636, 95% CI 1.056–2.535, *P* = 0.027), general anesthesia (OR 2.394, 95% CI 1.223–4.686, *P* = 0.011), and combined birthweight (OR 1.00032, 95% CI 1.00005–1.00059, *P* = 0.020). Collectively, in women with twin pregnancies delivered by cesarean section, the use of ART, preeclampsia or HELLP syndrome, placenta previa, placenta accreta, thrombocytopenia, general anesthesia and the combined birthweight were identified as independent risk factors for PPH. More attention should be paid to women with these risk factors.

## Introduction

The rate of twin pregnancies has obviously increased in recent years due to the advanced maternal age and the widespread use of assisted reproductive technology (ART) (1, 2). Cesarean section rate has also increased for the maternal fear of vaginal delivery failure and maternal preference for cesarean delivery, as well as the aversion to prolonged delivery (3).

Postpartum hemorrhage (PPH), one of the leading causes of maternal death, (4) is more likely to occur both in twin pregnancies (5, 6) and in cesarean deliveries (5). In twin gestations, the uterine overdistension is quite common and it is an important contributor to uterine atony (7). Increased maternal blood volume and uterine blood flow may be another potential explanation for the high risk of PPH in twin pregnancies (8). Moreover, in twin pregnancies, elective cesarean delivery has always been scheduled for fetal or maternal indications including placenta previa, placenta accreta and neonatal birthweight, as well as non-scheduled cesarean delivery has always been performed for fetal heart instability, umbilical cord prolapse, placental abruption and other emergency indications, which requires more strict clinical management to prevent PPH. Therefore, it is greatly important to identify the risk factors for PPH in twin pregnancies who delivered by cesarean section.

Several studies have been performed to explore the risk factors for PPH in twin deliveries but they have mainly focused on only one risk factor, and the mode of delivery includes both vaginal births and cesarean deliveries (9–11). A study led by Blitz et al. (8) has attempted to develop a predictive model for PPH in twin births, but they focused on PPH with packed red blood cells. To the best of our knowledge, there is limited data concentrating on the risk factors for PPH in twin-pregnant women who underwent cesarean section. Additionally, intertwin delivery interval (ITDI), defined as the interval time between the delivery of the first and the second twin, is rarely regarded as a potential risk for PPH by affecting uterine contractions.

Therefore, the purpose of our study was to identify the risk factors including ITDI for PPH among women with twin pregnancies during the cesarean section, which may provide recommendations on the optimal ITDI during cesarean section and improve the clinical management of twin pregnancies who were at high risk of PPH.

## Material and methods

### Study design, population and data collection

This was a retrospective observational study including all the twin-pregnant women giving birth after 24 weeks’ gestation by cesarean section in the Second Affiliated Hospital of Wenzhou Medical University from 1 January 2016, to 31 August 2022. Participants were eligible with diamniotic twin pregnancies (monochorionic or dichorionic diamniotic with two fetuses) and aged 18–46 years with complete medical records. Women with congenital uterine malformation, pre-pregnancy coagulation abnormalities, pre-pregnancy thyroid dysfunction, chronic hypertension or diabetes, chronic hepatic, renal or cardiac disease, immune-related diseases and those with single intrauterine fetal demise, multifetal pregnancy reduction, a combined mode of delivery (the first twin delivered by vaginal birth while the second twin delivered by cesarean section), fetal malformations including congenital heart disease, cleft lip and palate, congenital pulmonary airway malformations, anorectal malformations, and scoliosis/skeletal malformations, fetal chromosomal abnormalities and stillbirths were excluded. The eligible participants were further divided into PPH group and non-PPH group according to the blood loss after delivery within 24 h.

Medical records were reviewed to obtain baseline maternal and perinatal characteristics of the study population, including maternal age, gravidity, parity, uterine myoma, ART usage, gestational age at delivery, previous cesarean section, body mass index (BMI) at delivery, hypertensive disorders of pregnancy (HDP, including gestational hypertension and preeclampsia: gestational hypertension, defined as systolic blood pressure of 140 mmHg or more, or diastolic blood pressure of 90 mmHg or more, or both, after 20 gestational weeks; preeclampsia, defined as hypertension occurred after 20 gestational weeks combined with proteinuria, hemolysis or thrombocytopenia, renal or liver dysfunction, pulmonary edema, neurological features, or uteroplacental dysfunction), gestational diabetes mellitus (GDM, diagnosed by a 75-g oral glucose tolerance test conducted at 24 to 28 weeks of pregnancy: fasting glucose ≥ 5.1 mmol/L, or 1-h glucose ≥ 10.0 mmol/L, or 2-h glucose ≥ 8.5 mmol/L), HELLP syndrome (hemolysis, elevated liver enzymes, and low platelet), intrahepatic cholestasis of pregnancy (ICP, diagnosed by the elevated bile acids and pruritus), gestational hypothyroidism, placenta previa (including marginal, partial, and complete placenta previa), placental abruption, placenta accreta, low-lying placenta (the distance of placenta lower edge to cervical internal os is within 2 cm), premature rupture of membranes, chorioamnionitis, polyhydramnios (amniotic fluid index ≥ 24 cm or maximal vertical pocket of ≥ 8 cm), thrombocytopenia and general anesthesia. Data on unique characteristics of the twins were also collected, such as gestational age at delivery, twin-to-twin transfusion syndrome, combined birthweight of the twins, and the birthweight difference.

### PPH diagnosis

Postpartum hemorrhage (PPH) was defined as cumulative blood loss exceeding 1,000 ml after delivery according to the guidelines of ACOG (American College of Obstetrics and Gynecology) (12). In this study, quantitative blood loss measurement was calculated by weighing the surgical pad before and after surgery, or by multiplying the difference between the preoperative and postoperative hemoglobin values by the women’s estimated blood volume.

### Statistical analysis

All statistical analyses were generated using SPSS 25.0 software. Kolmogorov–Smirnov test was performed to determine distribution normality. Mean ± standard deviation (SD) and median (interquartile range, IQR) were used for the description of normally and non-normally continuous quantitative variables, respectively. Categorical data were described as percentages (%). Normally distributed values were compared using independent samples Student’s *t*-test, while the Mann–Whitney U test was utilized for non-normally distributed covariates. The fisher’s exact test and chi-squared test were used to analyze the differences between categorical variables.

Logistic regression analysis was performed to identify the potential risk factors for PPH in twin pregnancies who underwent cesarean section. Odds ratios (ORs), 95% confidence intervals (CIs) and *P*-values were calculated. Variables in the univariate analysis with *P*-values < 0.2 were selected as potential risk factors for PPH in the logistic regression. The goodness of fit was evaluated by the Hosmer–Lemeshow statistic. A *P*-value < 0.05 was considered to be statistically significant.

## Results

**FIGURE 1:**
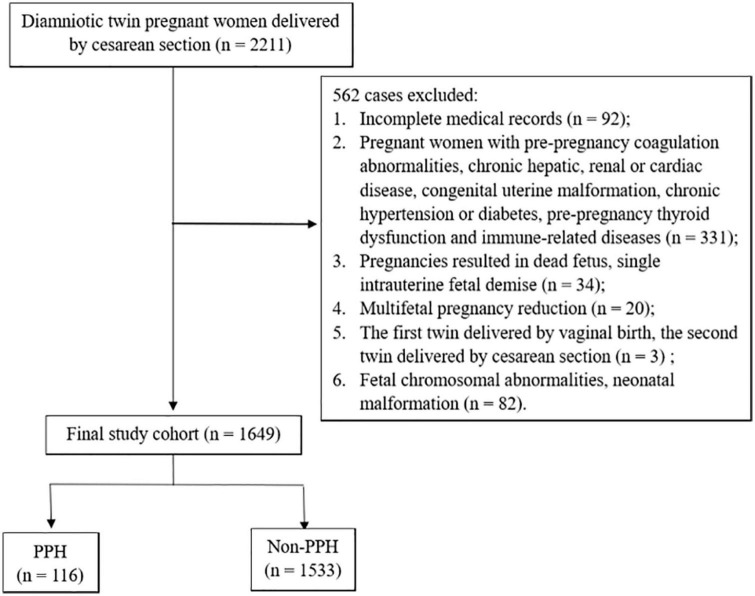
Selection of the study cohort (PPH, postpartum hemorrhage).

The selection of the study cohort was presented in Figure 1. A total of 2,211 pregnant women with diamniotic twins who gave birth by cesarean section in our institution were included from 1 January 2016, to 31 August 2022, among whom 562 women were excluded for the following reasons: (1) incomplete medical records (*n* = 92); (2) pregnant women with pre-pregnancy coagulation abnormalities, chronic hepatic, renal or cardiac disease, congenital uterine malformation, chronic hypertension or diabetes, pre-pregnancy thyroid dysfunction and immune-related diseases (*n* = 331); (3) pregnancies resulted in dead fetus, single intrauterine fetal demise (*n* = 34); (4) multifetal pregnancy reduction (*n* = 20); (5) the first twin delivered by vaginal birth, the second twin delivered by cesarean section (*n* = 3); (6) fetal chromosomal abnormalities, neonatal malformation (*n* = 82). Finally, 1,649 eligible women remained and were further divided into the PPH group (*n* = 116) and the non-PPH group (*n* = 1,533) according to the blood loss within 24 h after delivery.

The baseline and perinatal characteristics of the mothers in the PPH group and the non-PPH group were presented in Tables 1, 2. Little differences were found in maternal age, gravidity, previous cesarean section, monochorionicity, uterine myoma, gestational hypertension, GDM, ICP, gestational hypothyroidism, placental abruption, low-lying placenta, premature rupture of membranes, chorioamnionitis, polyhydramnios and thrombocytopenia between the two groups (*P* > 0.05). The rates of twin-to-twin transfusion syndrome, preterm delivery, very preterm delivery, gestational age at delivery, birthweight difference and ITDI also showed little differences between the PPH group and the non-PPH group (*P* > 0.05). However, nulliparity [88 (75.9%) vs. 978 (63.8%), *P* = 0.013), ART usage (94 (81.0%) vs. 924 (60.3%), *P* < 0.001], preeclampsia or HELLP syndrome [19 (16.4%) vs. 105 (6.8%), *P* < 0.001], placenta previa [14 (12.1%) vs. 36 (2.3%), *P* < 0.001], placenta accreta [3 (2.6%) vs. 6 (0.4%), *P* = 0.028], general anesthesia [12 (10.3%) vs. 74 (4.8%), *P* = 0.012] were significantly more common in the PPH group when compared to the non-PPH group. In addition, women in the PPH group had a significantly higher maternal BMI at delivery [28.31 (26.17–30.48) kg/m^2^ vs. 27.44 (25.48–29.74) kg/m^2^, *P* = 0.012] and a significantly higher combined birthweight of the twins as compared to the non-PPH group (5,002.54 ± 805.120 g vs. 4,821.17 ± 841.147 g, *P* = 0.023), but had lower parity [0 (0–0) vs. 0 (0–1), *P* = 0.01].

**TABLE 1:** Baseline maternal characteristics in the PPH group and the non-PPH group.

Characteristics	PPH (*n* = 116)	Non-PPH (*n* = 1,533)	*P*-value
Maternal age (years), mean ± SD	29.91 ± 3.914	30.19 ± 4.269	0.480
Gravidity, median (IQR)	2 (1–3)	2 (1–3)	0.653
Parity, median (IQR)	0 (0–0)	0 (0–1)	0.010
Nulliparous, *n* (%)	88 (75.9)	978 (63.8)	0.009
Previous cesarean section, *n* (%)	13 (11.2)	202 (13.2)	0.544
BMI at delivery (kg/m^2^), median (IQR)	28.31 (26.17–30.48)	27.44 (25.48–29.74)	0.009
The use of ART, *n* (%)	94 (81.0)	924 (60.3)	<0.001
Monochorionicity, *n* (%)	17 (14.7)	340 (22.2)	0.058

PPH, postpartum hemorrhage; SD, standard deviation; IQR, interquartile range; BMI, body mass index; ART, assisted reproductive technology.

**TABLE 2:** Clinical characteristics in the PPH group and the non-PPH group.

	PPH (*n* = 116)	Non-PPH (*n* = 1,533)	*P*-value
**Mothers**			
Uterine myoma, *n* (%)			0.315
Uterine myoma < 3 cm	4 (3.4)	101 (6.6)	
3 cm ≤ uterine myoma < 5 cm	0 (0)	24 (1.6)	
Uterine myoma ≥ 5 cm	0 (0)	11 (0.7)	
Gestational hypertension, *n* (%)	7 (6.0)	66 (4.3)	0.383
Preeclampsia or HELLP syndrome, *n* (%)	19 (16.4)	105 (6.8)	<0.001
Gestational diabetes mellitus, *n* (%)	25 (21.6)	313 (20.4)	0.770
ICP, *n* (%)	6 (5.2)	55 (3.6)	0.438
Gestational hypothyroidism, *n* (%)	5 (4.3)	49 (3.2)	0.425
Placenta previa, *n* (%)	14 (12.1)	36 (2.3)	<0.001
Placental abruption, *n* (%)	1 (0.9)	10 (0.7)	0.553
Placenta accreta, *n* (%)	3 (2.6)	6 (0.4)	0.021
Low-lying placenta, *n* (%)	4 (3.4)	27 (1.8)	0.271
Premature rupture of membranes, *n* (%)	15 (12.9)	236 (15.4)	0.476
Chorioamnionitis, *n* (%)	2 (1.7)	67 (4.4)	0.229
Polyhydramnios, *n* (%)	6 (5.2)	47 (3.1)	0.265
Thrombocytopenia, *n* (%)	34 (29.3)	337 (22.0)	0.068
General anesthesia, *n* (%)	12 (10.3)	74 (4.8)	0.010
**Neonates**			
Twin-to-twin transfusion syndrome, *n* (%)	1 (0.9)	4 (0.3)	0.306
ITDI, *n* (%)			0.658
0 min < ITDI ≤ 3 min	64 (55.2)	788 (51.4)	
3 min < ITDI ≤ 6 min	46 (39.7)	641 (41.8)	
6 min < ITDI ≤ 9 min	6 (5.2)	104 (6.8)	
Gestational age at delivery (weeks), median (IQR)	36.57 (35.14–37.11)	36.43 (35.14–37.00)	0.632
Preterm delivery (< 37 weeks), *n* (%)	74 (63.8)	1,051 (68.6)	0.288
Very preterm delivery (< 34 weeks), *n* (%)	10 (8.6)	183 (11.9)	0.284
Combined birthweight (g), mean ± SD	5,002.54 ± 805.120	4,821.17 ± 841.147	0.025
Birthweight difference (g), mean ± SD	250.73 ± 231.736	276.93 ± 251.886	0.278

PPH, postpartum hemorrhage; HELLP syndrome, hemolysis, elevated liver enzymes, and low platelet; ICP, intrahepatic cholestasis of pregnancy; ITDI, intertwin delivery interval; IQR, interquartile range; SD, standard deviation.

As shown in Table 3, a logistic regression model adjusting for nulliparous, parity, BMI at delivery, the use of ART, monochorionicity, preeclampsia or HELLP syndrome, placenta previa, placenta accreta, thrombocytopenia, general anesthesia and combined birthweight was performed. Although nulliparous, parity, BMI at delivery and monochorionicity were not associated with higher risks for PPH (*P* > 0.05), seven independent risk factors were identified: ART usage (OR 2.354 95% CI 1.357–4.083, *P* = 0.002), preeclampsia or HELLP syndrome (OR 2.605, 95% CI 1.471–4.616, *P* = 0.001), placenta previa (OR 7.325, 95% CI 3.651–14.697, *P* < 0.001), placenta accreta (OR 6.296, 95% CI 1.316–30.12, *P* = 0.021), thrombocytopenia (OR 1.636, 95% CI 1.056–2.535, *P* = 0.027), general anesthesia (OR 2.394, 95% CI 1.223–4.686, *P* = 0.011), and combined birthweight (OR 1.00032, 95% CI 1.00005–1.00059, *P* = 0.020).

**TABLE 3:** Logistic regression model analysis of the potential risk factors for PPH in in twin pregnancies delivered by cesarean section.

Potential risk factors	OR (95% CI)	*P*-value
**Mothers**		
Nulliparous	1.700 (0.482–5.997)	0.410
Parity	1.087 (0.390–3.035)	0.873
BMI at delivery	1.038 (0.981–1.098)	0.194
The use of ART	2.354 (1.357–4.083)	0.002
Monochorionicity	0.827 (0.450–1.519)	0.539
Preeclampsia or HELLP syndrome	2.605 (1.471–4.616)	0.001
Placenta previa	7.325 (3.651–14.697)	< 0.001
Placenta accreta	6.296 (1.316–30.122)	0.021
Thrombocytopenia	1.636 (1.056–2.535)	0.027
General anesthesia	2.394 (1.223–4.686)	0.011
**Neonates**		
Combined birthweight	1.00032 (1.00005–1.00059)	0.020

PPH, postpartum hemorrhage; OR, odds ratio; CI, confidence intervals; BMI, body mass index; ART, assisted reproductive technology; HELLP syndrome, hemolysis, elevated liver enzymes, and low platelet.

## Discussion

In this retrospective observational study, the results demonstrated that the use of ART, preeclampsia or HELLP syndrome, placenta previa, placenta accreta, thrombocytopenia, general anesthesia and the combined birthweight were independent risk factors for PPH among women with twin pregnancies who delivered by cesarean section, while ITDI was not.

Our findings support certain factors previously identified to be associated with PPH in twin pregnancies, while cast doubt on others. Most prior investigators have reported that twin gestations conceived by ART are associated with a significantly increased rate of PPH when compared to spontaneous conception, which is in consistent with our findings (13, 14). This might be because ART usage increases the potential risk factors for PPH including placenta accreta spectrum, (13) placental abruption and gestational hypertension (15). Consequently, it is advisable to avoid multiple or twin pregnancies in ART procedures. However, another study led by Pourali et al. (9) found little difference in the incidence of PPH between ART-conceived pregnancies and spontaneous-conceived pregnancies. The discrepancy of the results may be associated with the different population and region, as well as the different sample size and study methods. For twin pregnancies conceived by ART who delivered by cesarean section, more attention should be paid to prevent postpartum bleeding.

There are four types of HDP including chronic hypertension, preeclampsia, gestational hypertension, and superimposed preeclampsia (16). In our study, women with chronic hypertension and superimposed preeclampsia were excluded. Preeclampsia or HELLP syndrome was found to be a risk factor for PPH in twin pregnant women who underwent cesarean section. Similar to the results of our study, previous studies have also identified preeclampsia as a risk factor for PPH (5, 6) and HELLP syndrome has been proved to be related to a higher risk of PPH with transfusion (17). However, gestational hypertension in our study showed little association with PPH, which was in accordance with a previous research conducted in China (5). A possible explanation may be that mild HDP may not be a contributor to PPH. Impaired spiral arteriole remodeling, immunologic abnormalities and endothelial dysfunction are associated with the severity of preeclampsia, and these pathological changes can be found in severe HDP but may not be found in mild HDP. Therefore, it is important to prevent and control the occurrence and development of preeclampsia or HELLP syndrome in women with twins.

One research compared the guidelines of the prevention and management of PPH among four countries and identified placenta accreta, placenta previa or low-lying placenta, placental abruption as high risk factors for PPH (18). Similarly, placenta previa and placenta accreta were considered to be two main independent risk factors for PPH in this study, by approximately 3.651 to 14.697 fold risk in the former and 1.316 to 30.122 fold risk in the latter, as compared to the women without these complications after adjusting the potential risk factors for PPH. However, we found little difference in the rates of low-lying placenta and placental abruption between the PPH group and non-PPH group. This might be mainly accounted by the different characteristics of the placenta and the location of incisions on the uterus during operation. For example, there were more abundant vasculature existing in the sub-placental zone and the lower placental edge, and there were more blood vessels at the incision of the anterior placenta than the posterior placenta. To avoid transplacental delivery of the fetus and avoid areas full of blood vessels, a relative high transverse incision might be adopted, so the uterine wall in the incision was thicker, resulting in more bleeding. A literature has reported that women with placenta reaching/covering os, anterior placenta and thick placenta are more likely to develop massive hemorrhage after cesarean section, while those with a distance from the placental edge to os of 0–10 mm, 10–20 mm shows little association with severe PPH (19). These findings confirmed the importance of antenatal assessment of placenta previa and placenta accreta, and also emphasized the necessary of transference to a tertiary care center with rapid access to blood products or an intensive care unit.

Approximately 22.5% of women in our cohort had gestational thrombocytopenia, which was greatly higher than the commonly reported rate of thrombocytopenia in the general obstetric population (20). This may be explained by the population characteristics and it has been reported that thrombocytopenia is more likely to occur in twin gestations than singletons (21). The increased incidence of thrombocytopenia in twin pregnancies may be attributed to the enhanced platelet reactivity, (21) greater hemodilution (8) as compared to singleton pregnancies, or both. A study of 1,085 women with twin gestations found a greater incidence of PPH in women with mild thrombocytopenia compared with those with normal platelet count (10). Additionally, moderate thrombocytopenia (22) and severe thrombocytopenia (8) were also convinced to be risk factors for PPH in twin pregnancies. Based on these findings, (8, 10, 22) a conclusion that thrombocytopenia in pregnancy was associated with PPH regardless of its severity could be deduced. In addition, our study demonstrated that gestational thrombocytopenia was indeed more common in women with PPH. Collectively, it is of great importance to pay attention to women with thrombocytopenia for the prevention of PPH.

Our study showed that the odds of PPH was 2.394-fold higher for twin-pregnant women who received general anesthesia as compared to those who received spinal or epidural anesthesia. This result was similar to previous findings that women with general anesthesia during cesarean delivery were more likely to experience PPH (18, 23). The increased incidence of pregnancy complications, such as preeclampsia or HELLP syndrome, placenta previa and placenta accreta, may be attributed to the administration of general anesthesia in women with cesarean section. It has been reported by several studies that most of these complications are associated with PPH (5, 6). Additionally, it has been demonstrated that several drugs used for general anesthesia can exert a suppressive effect on uterine contraction (23). The property of suppressing uterine contraction, platelet function and hemostasis of general anesthetics may lead to the higher risk of PPH (23). Therefore, the decision to use general anesthesia should be carefully made when the formulation of anesthesia protocols is planned for hemorrhage-prone women.

The result in our study found that the incidence of PPH was gradually increased with the combined birthweight, which was in accordance with the study led by Loussert et al. (11). This is probably due to the fact that the high combined birthweight of the twins is related to the overstretched uterus, thus affecting uterine contractions. On the contrary, another study conducted by Blitz et al. (8) found little association between the combined birthweight and PPH (8). The different study design and the different definition of PPH could partially explain the contrast results.

Data concentrating on the relationship between ITDI and PPH are limited. We included ITDI as a potential risk of PPH for the following reasons. First, we postulated that appropriately prolonging the delivery of the second twin might potentially decrease the incidence of PPH by facilitating uterine packing and inducing uterine contractions. Second, by properly increasing the interval between births of twins, we can prevent sudden decompression of the uterus after rupture of the fetal membrane, thereby reducing complications such as placental abruption. Contrary to our hypothesis, there were little differences in ITDI and the rate of placental abruption between the PPH group and the non-PPH group. This might be because once PPH occurs, the effect of uterine packing and uterine contraction induced by appropriately prolonged ITDI seemed to be useless. And the association between ITDI and blood loss still needs to be further explored.

## Strengths and limitations

To the best of our knowledge, limited research was conducted to explore the risk factors for PPH in twin pregnancies with cesarean section and we firstly included ITDI as a potential risk factor. By improving the identification of those with high risk of PPH may help clinicians optimize delivery plans, promote early and optimal management of PPH and finally prevent the further aggravation of PPH. Another strength of our study is the relatively large sample size that allow a proper statistical analysis. However, the retrospective observational research design was our limitation.

## Conclusion

In women with twin pregnancies underwent cesarean section, the use of ART, preeclampsia or HELLP syndrome, placenta previa, placenta accreta, thrombocytopenia, general anesthesia and the combined birthweight were identified as independent risk factors for PPH, while ITDI was not. For women with ART usage, preeclampsia or HELLP syndrome, placenta previa, placenta accreta, thrombocytopenia and high combined birthweight, advanced delivery planning, experienced clinicians, appropriate level of maternal care should be offered. General anesthesia should be applied cautiously.

## Data availability statement

The raw data supporting the conclusions of this article will be made available by the authors, without undue reservation.

## Ethics statement

The studies involving humans were approved by the Research Ethics Committee of the Second Affiliated Hospital of Wenzhou Medical University (approval number: 2022-K-139-01). The studies were conducted in accordance with the local legislation and institutional requirements. The participants provided their written informed consent to participate in this study.

## Author contributions

YL: Writing – original draft. AX: Writing – review & editing. XL: Writing – review & editing. YZ: Writing – review & editing, Data curation. JW: Writing – review & editing. YH: Writing – review & editing, Funding acquisition, Supervision. KD: Writing – review & editing, Funding acquisition, Supervision.
